# Total *en Bloc* Urethra Preservation at Robot-Assisted Prostatectomy (ToP-RAP): A Prospective IDEAL Stage 1–2a Study

**DOI:** 10.3390/cancers18193134

**Published:** 2026-09-28

**Authors:** Mahmoud Farzat, Mykyta Kachanov, Malte W. Vetterlein, Georg Salomon, Ibrahim Al Taie, Su Jung Oh-Hohenhorst, Florian M. Wagenlehner, Hubert John, Jorn H. Witt, Sami-Ramzi Leyh-Bannurah

**Affiliations:** 1 Department of Urology and Robotic Urology, Diakonie Klinikum Siegen, 53127 Siegen, Germany; mahmoud.farzat@diakonie-sw.de (M.F.); ibrahim.al-taie@diakonie-sw.de (I.A.T.); 2 Department of Urology, Pediatric Urology and Andrology, Justus-Liebig University of Giessen, 35390 Giessen, Germany; florian.wagenlehner@chiru.med.uni-giessen.de; 3 Department of Urology, University Medical Center Hamburg-Eppendorf, 20251 Hamburg, Germany; kachanovn@gmail.com (M.K.); m.vetterlein@uke.de (M.W.V.); s.oh-hohenhorst@uke.de (S.J.O.-H.); 4 Martini-Klinik, Prostate Cancer Center, University Medical Center Hamburg-Eppendorf, 20246 Hamburg, Germany; g.salomon@uke.de; 5 Department of Urology, Winterthur Kantonsspital, 8401 Winterthur, Switzerland; hubert.john@ksw.ch; 6 Department of Urology and Urologic Surgery, University Medical Center Mannheim, University of Heidelberg, 68167 Mannheim, Germany; jhwitt@urokompetenz.de; 7 Department of Urology, Urosurgery and Robotic Surgery, Clinic Bel Etage, 40217 Dusseldorf, Germany; 8 Prostate Center Gronau, Department of Urology, Uro-Oncology and Robot-Assisted Urology, St. Antonius-Hospital, 48599 Gronau, Germany

**Keywords:** prostatic neoplasms, minimally invasive surgical procedures, robotic surgical procedures, prostatectomy, urinary incontinence, feasibility studies

## Abstract

**Background/objective:** Urinary continence recovery after robot-assisted radical prostatectomy remains variable despite technical refinements intended to preserve function. We describe Total *en bloc* Urethra Preservation at Robot-assisted Prostatectomy (ToP-RAP), a technique that preserves the entire intraprostatic urethra and avoids urethral transection, and report short-term functional and oncological outcomes. **Methods:** Patients with PSA ≤ 15 ng/mL, prostate volume ≤ 80 mL, biopsy ISUP grade ≤ 3, and predefined selection criteria underwent surgery in this single-arm, two-center, IDEAL stage 1–2a development study (ClinicalTrials.gov NCT07678489). Surgical, perioperative safety, continence, and oncological outcomes based on PSA, imaging, and secondary treatment were assessed descriptively. **Results:** The first 16 consecutive patients were treated between 02/2024 and 05/2025. Median age was 66 years (IQR 62–71), PSA was 6.7 ng/mL (IQR 4.7–9.6), and prostate volume was 33 mL (IQR 29–46). Biopsy ISUP grades 1, 2, 3 were in 19%, 44%, and 38% of patients, respectively. On MRI, 38% of patients had multifocal bilateral lesions. Urethral repair of ≤1 cm was required in 13% of patients. All patients underwent at least interfascial nerve sparing. One posterolateral positive surgical margin and one perioperative complication (fascial dehiscence) occurred, no further complications were observed within 90 days. Median follow-up was 7.2 months (IQR 6.1–10.7). No patient had PSA persistence, biochemical recurrence, or secondary treatment. Overall, 15 of 16 patients (94%) recovered urinary continence, and 94% did so within 5 days. Limitations include the small cohort and short follow-up. **Conclusions:** Early findings support the feasibility and short-term safety of ToP-RAP. The observed early continence recovery warrants evaluation in larger cohorts with longer follow-up.

## Simple Summary

After prostate cancer surgery, some men may have urinary leakage that can strongly affect daily life, even when modern robotic techniques are used. This study introduces a new robotic surgical approach, Total *en bloc* Urethra Preservation at Robot-assisted Prostatectomy, designed to keep the entire urine channel inside the prostate intact and avoid cutting and reconnecting it. The authors aimed to test whether this technique can be performed safely in carefully selected men with localized prostate cancer and whether it may allow faster recovery of urinary control without early signs of reduced cancer control. In the first 16 patients, 15 regained urinary control, most within a few days, and no early cancer recurrence or additional treatment was observed during short follow-up. These early results may guide further research into function-preserving prostate cancer surgery, but larger studies with longer follow-up are needed.

## 1. Introduction

Robot-assisted radical prostatectomy (RARP) is the standard treatment for localized, clinically significant prostate cancer (PCa). Compared with open radical prostatectomy, the proposed benefits of RARP include lower intraoperative blood loss, smaller incisions, faster recovery, and shorter hospital stay [1,2]. Nonetheless, post-prostatectomy urinary incontinence remains a clinically significant adverse outcome, with reported rates of up to 30% [3].

Urinary incontinence after RARP is complex and multifactorial, with contributing mechanisms including intrinsic sphincter deficiency, loss of urethral support, and alterations of the bladder neck or anastomotic configuration. Of particular importance are the functional length and support of the membranous urethra and the external urethral sphincter. Even complications such as urinary extravasation may lead to secondary fibrosis at the anastomosis, potentially compromising the continence mechanism through distortion of the urethral axis or impaired coaptation [4].

Early return of continence is a principal determinant of postoperative patient satisfaction and quality of life; even mild stress urinary incontinence can adversely affect social and sexual functioning [5]. Accordingly, there is a strong impetus to optimize both the rate and the timing of complete continence recovery while minimizing urinary complications [6]. In context of adequate patient selection, molecular markers such as aneuploidy and its driver genes stratify progression and prognosis of prostate cancer [7] and may in future refine the imaging- and biopsy-based selection for urethra-preserving surgery. For these reasons, some PCa patients opt for focal therapy (FT), primarily to preserve quality of life and avoid treatment-related morbidity while maintaining oncological control [8]. FT typically involves targeting the cancerous lesion(s) within the prostate while sparing the surrounding healthy tissue and critical structures involved in urinary continence and sexual function [9].

Based on similar principles, we propose a robotic FT that preserves the entire intraprostatic urethra, thereby avoiding urethral transection and vesicourethral anastomosis while preserving the adjacent neurovascular bundles with real-time frozen section analysis. Within the five-stage IDEAL (Idea, Development, Exploration, Assessment, Long-term study) framework [10,11], we conducted a prospective, multicenter stage 1–2a/b study of a novel technique termed Total *en bloc* Urethra Preservation during Robot-Assisted Prostatectomy (ToP-RAP), aiming to combine and extend previous advances in functional preservation without compromising oncological principles.

## 2. Materials and Methods

### 2.1. Study Design

The present investigation was conducted as a single-arm, multicenter, prospective IDEAL stage 1–2a/b development study in accordance with the methodological principles of the IDEAL framework for surgical innovation [10,11]. Following histologically confirmed PCa, eligible patients were invited to participate at two centers (Diakonie Klinikum Jung-Stilling, Siegen, Germany, and Clinic Bel Etage, Düsseldorf, Germany).

All patients underwent detailed preoperative counseling, including a comprehensive explanation of the surgical procedure. This counseling highlighted the differences between ToP-RAP and standard RARP, as well as potential risks, benefits, functional and oncological outcomes, and possible retreatment options. Patients who did not meet the inclusion criteria or declined participation were offered standard RARP. Written informed consent was obtained from all participants prior to enrollment.

For each IDEAL stage 1 patient, the Chief of the Department of Urology, who performed the procedure, obtained written informed consent that disclosed the novel, first-in-human nature of the technique, its anticipated and unknown risks, and the available alternatives. The prospective stage 2a protocol was approved by the Ethics Committees of the Medical Associations Westphalia-Lippe and North Rhine, 28 March 2025 and 14 July 2025, respectively. The study is registered with ClinicalTrials.gov, 20 June 2026 (NCT07678489). Registration was retrospective relative to the start of enrolment; it was completed when the stage 2a cohort was consolidated for analysis. No primary or secondary outcome was changed after enrolment of the first participant.

Clinical data acquisition, processing, and storage were conducted in accordance with the European General Data Protection Regulation (EU 2016/679). Follow-up assessments were scheduled at 3, 6, and 12 months postoperatively.

### 2.2. Patient Selection

A total of 47 patients were enrolled within the IDEAL framework (stage 1: n = 5; stage 2a/b: n = 42) between February 2024 and December 2025. Analyses were restricted to patients undergoing ToP-RAP with at least 6 months of follow-up (n = 16) (Supplementary Figure S1).

All procedures were performed by two experienced robotic surgeons (M.F. and J.H.W.), each with ≥2500 robotic procedures—including RARP—using the da Vinci X/Xi^®^ platforms (Intuitive Surgical Inc., Sunnyvale, CA, USA).

Patients were eligible if they had a life expectancy of ≥10 years based on biological age, and no history of prior prostate surgery or radiotherapy (e.g., laser enucleation, transurethral resection of the prostate, or FT), prior rectal surgery, prior androgen deprivation therapy, or cerebrovascular or neurological disease (e.g., Parkinson’s disease).

Clinical prerequisites included transrectal or transperineal MRI–ultrasound fusion-guided biopsy with combined targeted and systematic sampling comprising at least 12 cores. In men with International Society of Urological Pathology (ISUP) grade 1 disease, systematic biopsy alone was considered sufficient, and prostate MRI was optional. When eligible and appropriately counseled regarding all treatment options—including active surveillance—the decision to undergo ToP-RAP was initiated by the patient.

Inclusion criteria comprised prostate-specific antigen (PSA) ≤ 15 ng/mL, prostate volume ≤ 80 mL, and biopsy ISUP grade 1–3, with absence of Gleason pattern 5, intraductal, cribriform, or neuroendocrine carcinoma. If a lesion was visible on bi- or multiparametric MRI (mpMRI), it had to be predominantly located in the peripheral zone, without evidence of extracapsular extension, anterior midline involvement, seminal vesicle invasion, or unequivocal metastatic disease on imaging. Patients with presence of a median lobe > 1.0 cm were excluded. Median lobe protrusion was assessed preoperatively using mid-sagittal T2-weighted MRI (otherwise TRUS if MRI was not available) and measured from the bladder-base line to the tip of the intravesical prostatic component; patients with median lobe protrusion >1.0 cm were excluded. Intravesical prostatic protrusion was graded as grade I (<5 mm), grade II (5–10 mm), or grade III (>10 mm).

### 2.3. Surgical Technique

A standard transperitoneal RARP approach was used. Pelvic lymph node dissection was performed [12].

At this transition zone, the urethra was partially released from the prostatic base by blunt dissection [12]. The vesicoprostatic muscle was then dissected paramedially, immediately adjacent to the intact bladder neck, until the right seminal vesicle was visualized (Figure 1), after which the ipsilateral neurovascular bundle was carefully released in an anterolateral fashion from base to apex. Following neurovascular bundle release, apical urethral preparation was performed, enabling complete functional-length urethral sphincter preservation [13]. This was followed by selective ligation of the dorsal venous complex. At this point, the prostate was fully mobilized anteriorly, laterally, and posteriorly, remaining attached only to the intact prostatic urethra.

The anterior aspect of the prostate was then incised in the midline until the longitudinal urethral fibers were exposed (“open book maneuver”), spreading both lateral lobes laterally with the urethra in the midline (Figure 2a,b). The prostatic urethra was bluntly dissected from the lateral lobes along its entire length and circumference. The central base segment, containing the ejaculatory ducts, typically required sharp incision. In the event of small urethral defects ≤ 1 cm, primary watertight repair with absorbable suture was performed.

After complete prostatic release, the specimen was extracted and processed for intraoperative frozen section analysis of both the neurovascular bundle-adjacent posterolateral aspect of the prostate and the prostatic tissue surrounding the intraprostatic urethra. A bladder filling test was performed to identify urethral leakage. Adequate urethral vascularity was verified by intravenous administration of indocyanine green (ICG; VERDYE^®^, Diagnostic Green GmbH, Aschheim-Dornach, Germany) with near-infrared fluorescence using the Firefly^®^ system (Intuitive Surgical, Inc., Sunnyvale, CA, USA) [14]. ICG was reconstituted at the standard concentration of 2.5 mg/mL (25 mg/10 mL saline), and differential fluorescence was typically observed within 2 min of administration (Figure 3). In the event of deficient vascularity or complete urethral disruption, the urethra was resected and a standard vesicourethral anastomosis was fashioned, effectively converting the procedure to standard RARP.

Optionally a ventral reconstruction between the puboprostatic ligaments and the anterior periprostatic tissue and ventral bladder wall was performed resulting in extraperitonealization of the urethra and the prostatic bed. Patients received a suprapubic catheter, a transurethral catheter was retained for 24 h.

### 2.4. Postoperative Protocol

The transurethral catheter was removed within 48 h after surgery. The suprapubic catheter was clamped on postoperative day 3 or 4, and patients were permitted to void spontaneously. When micturition was uneventful for at least 24 h, the suprapubic catheter was removed the following day and patients were discharged. In cases of voiding dysfunction, defined as urinary retention with post-void residual urine > 150 mL, another 24 h observation period was allowed before repeating a voiding trial. For patients achieving a complete PSA response (PSA ≤ 0.1 ng/mL), biochemical recurrence (BCR) was defined as PSA > 0.2 ng/mL or a continuous rise over two consecutive measurements. For patients reaching a PSA nadir without achieving undetectable PSA levels, BCR was defined as a continuous rise over two consecutive measurements. Such patients underwent prostate-specific membrane antigen (PSMA) PET-CT. In cases of isolated tracer uptake in the prostatic region suggesting local recurrence, pelvic MRI was planned and patients were referred for adjuvant external beam radiotherapy of the prostatic bed per European Association of Urology (EAU) guidelines [15].

### 2.5. Endpoints and Covariates

The primary endpoints of this IDEAL stage 1–2a/b development study were to evaluate the safety, feasibility, and early functional recovery following ToP-RAP [10,11]. Safety outcomes included intraoperative and perioperative complication rates, assessed from the day of surgery through hospital discharge. Specific intraoperative events of interest included the need for urethral suturing or conversion to standard RARP. Specimens were fully fixed, inked with multiple colors for right/left and anterior/posterior orientation, and processed per International Society of Urological Pathology (ISUP) and International Collaboration on Cancer Reporting (ICCR) recommendations, with the apex and base examined by the modified cone method and the gland serially sectioned at 3–4 mm. Twenty intraoperative frozen-section samples were obtained per case: bilateral posterolateral (neurovascular-bundle-adjacent) sampling at apical, middle and basal levels (6 samples); circumferential urethral sampling as anterior and posterior hemi-circumferences at apical, middle and basal levels (6 samples); apical margins (ventral and dorsal, bilateral; 4 samples); and bladder-neck margins (ventral and dorsal, bilateral; 4 samples). Intraoperative apical tissue was reconciled with, not substituted for, the final cone-method apical margin. Preserved intracorporeal urethral length was measured intraoperatively. Per the ICCR dataset, final margins were reported using the required elements of margin status (involved/not involved) and location, linear extent (in millimeters) and Gleason pattern at the margin. Postoperative morbidity was assessed using the maximum Clavien–Dindo complication grade recorded within 90 days after surgery.

Functional recovery endpoints focused on urinary outcomes. Time to continence was calculated from the day of surgery to the first documentation of the defined continence state; because the first assessment coincided with catheter removal (day 4–5), this corresponds to time to catheter removal for the perioperative cohort [16,17]. Our first assessment was performed 24 h after catheter removal and subsequently at 3, 6, and 12 months (annually afterwards) postoperatively. Continence was assessed prospectively by a structured physician interview grading stress urinary incontinence by the Stamey criteria (severity grades 1–3; convergent validity with the ICIQ-UI SF up to ρ 0.92–0.95 after radical prostatectomy) [18,19], patient-reported daily pad use (none/one safety pad/one pad/two pads/≥ three pads) and a single leakage-frequency item corresponding to Question 1 of the ICIQ-UI Short Form (4-week recall) [20,21,22]. Lower urinary tract symptoms were assessed with the IPSS, reported here as a voiding-symptom measure [23,24,25].

Oncological control was assessed by serial PSA measurements obtained at 3, 6, 9, 12, 18, 24 months postoperatively (annually afterwards). BCR was defined as PSA ≥ 0.2 ng/mL; PSA persistence was defined as failure to achieve PSA ≤ 0.1 ng/mL within 6–12 weeks postoperatively.

### 2.6. Statistical Analyses

Descriptive statistics included frequencies and proportions for categorical variables, and medians with interquartile ranges (IQR) for continuous variables. Chi-squared tests were used for categorical variables and *t*-tests for continuous variables. All tests were two-sided, with *p* < 0.05 considered statistically significant. Analyses were performed using R, version 4.5.2 (R Foundation for Statistical Computing, Vienna, Austria).

## 3. Results

### 3.1. Baseline Clinical Characteristics

The total cohort comprised 47 PCa patients who fulfilled the study criteria and consented to ToP-RAP. Of these, eight patients were converted to standard RARP: urethral preservation was unsuccessful in three patients due to adherent tissue, in two due to median lobe anatomy, and three had positive urethral surgical margins on intraoperative frozen section examination. Specifically, the three patients converted to standard RARP for intraoperative urethral-margin positivity all achieved final R0 status; after successful ToP-RAP in the remaining 39 patients, restricting the cohort to those with at least 6 months of follow-up yielded 16 patients. The remaining 23 patients are actively followed up (Supplementary Figure S1).

Median age was 66 years (IQR 62–71) and median BMI was 27.6 kg/m^2^ (IQR 26–32; Table 1). Median preoperative PSA was 6.7 ng/mL (IQR 4.7–9.6), and median prostate volume was 33 mL (IQR 29–46). Clinical stage was cT1c in 69% of patients. Preoperative MRI demonstrated PI-RADS 3 lesions in 13%, PI-RADS 4 in 56%, and PI-RADS 5 in 19% of patients. Overall, 38% of patients harbored multifocal, bilateral disease. Biopsy ISUP grade was 1 in 19%, 2 in 44%, and 3 in 38% of patients.

### 3.2. Intra-Operative Outcomes

Median console time was 120 min (IQR 100–180; Table 2). Median estimated blood loss was 250 mL (IQR 200–300). Urethral suturing for a defect ≤1 cm was required in 13% of patients. All patients received grade 1 (complete intrafascial) or grade 2 (interfascial) nerve-sparing [26]. Final pathology ISUP grade was 1 in 13%, 2 in 56%, and 3 in 31% of patients. Final pathological stage was pT2 in 81%, pT3a in 13%, and pT3b in 6.3% of patients. All patients underwent pelvic lymph node dissection (PLND) at the time of surgery. In accordance with institutional policy, patients staged without PSMA-PET/CT undergo PLND to ensure accurate pathological nodal staging and to avoid nodal understaging. No lymph node metastases were identified and were classified pN0 accordingly. A posterolateral positive surgical margin was observed in 6.3% (n = 1); the surgical margin was negative on frozen section and positive on final pathology. One perioperative complication occurred (Clavien–Dindo grade 3b): fascial dehiscence requiring closure. No other postoperative complications were recorded within 90 days.

### 3.3. Follow-Up

Median total follow-up was 7.2 months (IQR 6.1–10.7). Median preoperative PSA was 6.7 ng/mL (range 3.0–12.0) and median postoperative PSA was 0.020 ng/mL (range 0.007–0.080; Supplementary Figure S2). Using the definitions applied throughout this report (i.e., PSA persistence as failure of PSA to fall below 0.1 ng/mL, and biochemical recurrence as PSA ≥ 0.2 ng/mL confirmed by a subsequent value > 0.2 ng/mL) no patient met criteria for PSA persistence (0/16) or biochemical recurrence (0/16); all patients achieved a conventional undetectable nadir (<0.1 ng/mL). Six of 16 patients (37.5%) recorded at least one value in the 0.05–0.08 ng/mL ultrasensitive range (highest observed value 0.08 ng/mL, in two patients); where serial data existed these values fluctuated without a sustained rise. Because urethra-preserving dissection may leave benign residual prostatic glandular tissue, which is a recognized source of low, stable ultrasensitive PSA, and because periurethral glands themselves do not materially raise serum PSA, these low-level values are most consistent with residual benign prostatic tissue rather than biochemical recurrence; conventional complete-extirpation thresholds should therefore be applied cautiously in the very low range. No patient required additional therapy during the observation period.

Over the total observation period, strict pad-free continence was achieved in 12 of 16 patients (75%), and social continence (0–1 safety pad) in 15 of 16 (94%). At catheter removal (day 3–6), 14 of 16 patients (87.5%) had a Stamey grade-0 assessment, and one additional patient was first documented continent at day 88, yielding 15 of 16 (94%) continent at earliest documentation, with a median time to first documented continence of 5 days (IQR 4–6). One patient did not achieve continence under either definition (≥3 pads/day, Stamey grade 3, leakage “more than once a day” at his only assessment, day 135 after surgery). His pattern was irritative rather than sphincteric (IPSS increased from 14 preoperatively to 23 post-ToP-RAP; voiding subscore 11 → 15; storage subscore 3 → 6; post-ToP-RAP post-void residual 0 mL), indicative of an urge-predominant, dysfunctional-voiding mechanism. No patient required re-catheterization or clean intermittent self-catheterization, and in the patients with postoperative IPSS data the maximum voiding subscore was 3, providing no evidence of de novo retention in the assessed subset.

## 4. Discussion

Several technical modifications of RARP have been reported to improve UCR, including the hood and sleeve techniques [27,28] and the Retzius-sparing approach [29], which demonstrated UCR rates of 91% at 3 months (hood technique) and 95% vs. 86% at 3 months (Retzius-sparing vs. standard RARP), respectively. Schlomm et al. identified complete functional-length urethral sphincter preservation as a crucial determinant of UCR [13]. Comparable outcomes were subsequently reported in robotic series using a distal verumontanum incision and bladder-neck preservation to maintain the internal sphincter [5,30]. A technique fairly close to ToP-RAP is complete urethral preservation (CUP) by Al-Hammouri et al., which preserves the prostatic urethra to the proximal verumontanum, transects it there and restores continuity by an end-to-end anastomosis [31]. ToP-RAP preserves the intraprostatic urethra in continuity and needs no anastomosis. CUP reported immediate zero-pad continence in 53% and 94% pad-free at 12 months, with 22.6% positive margins and 11.3% biochemical recurrence at 1 year in a cohort including 35% Cambridge Prognostic Group 4–5. Whether ToP-RAP’s omission of the anastomosis translates into fewer strictures and leaks and faster urinary continence than CUP can only be tested in a future comparative study. Feasibility of urethra-sparing dissection in a robotic setting has also been demonstrated in the context of benign prostatic hyperplasia by Simone et al. [32] and Porpiglia et al., who reported full continence at 12 months [33].

Despite these technical advances, UCR rates after RP remain markedly inferior to those after radiotherapy across multiple randomized trials. In PACE-A, 50% of prostatectomy patients versus 6.5% of SBRT patients used one or more pads per day at 2 years [34].

Similarly, in ProtecT, 46% of men reported pad use at 6 months after RP, decreasing to 17% at 6 years, with no change after radiotherapy [35]. Continence definitions and time points shift reported rates by 20–30 percentage points [36,37]; we therefore report both definitions and compare only with series using the same definition and time point.

Against this background, FT of the prostate is regarded as an attractive treatment option for patients with localized, MRI-visible, biopsy-confirmed PCa, because selective ablation of the cancerous region(s) spares the surrounding prostatic tissue and critical functional structures. Accordingly, FT yields high UCR rates (94–100% pad-free) with substantially preserved erectile function sufficient for intercourse (47–86%) and low rates of major complications [8,38,39]. We therefore aimed to combine the functional advantages FT with the aforementioned technical advancements within the IDEAL framework [8,10,11].

Our study has several important findings. First, consistent with FT consensus criteria [40], we applied stringent patient selection focusing on low- to intermediate-risk PCa. As a study prerequisite, patient evaluation relied on multiparametric MRI according to PI-RADS v2 and MRI–ultrasound fusion-guided targeted biopsies, providing comprehensive preoperative characterization of PCa including tumor size, intraprostatic localization, and probability of extracapsular extension and seminal vesicle invasion [41,42].

Second, despite the early-stage learning curve, surgical parameters—including pneumoperitoneum pressure, console time, and estimated blood loss—did not differ substantially from those of standard RARP. These findings suggest that the anatomical overlap between ToP-RAP and standard RARP enables expert surgeons to transfer their robotic skills efficiently.

Third, a similar observation was made regarding the intraoperative frozen section protocol, which was extended to include the intraprostatic urethral surface. The additional waiting time for the frozen section result was approximately 7 min—a negligible interval, particularly when pelvic lymph node dissection is performed concurrently. The single posterolateral false-negative frozen section is consistent with the 2.5–4.6% false-negative rate of NeuroSAFE [43].

Fourth, the single Clavien–Dindo grade 3b complication (fascial dehiscence requiring closure) represents a general surgical complication; no short-term complications specifically attributable to ToP-RAP were observed.

Fifth, small posterior urethral defects were readily amenable to immediate watertight suture repair. Notably, electrocautery was not employed during the meticulous dissection and release of the intraprostatic urethra, and intraoperative ICG fluorescence confirmed adequate urethral vascularity. These technical measures may contribute to long-term preservation of urethral function and potentially reduce the incidence of urethral strictures. In accordance with the IDEAL framework, no numerical threshold for the conversion rate was required to be prespecified for the stage 1–2a study. However, our protocol did predefine intraoperative conversion rules, i.e., positive periurethral frozen section, insufficient indocyanine-green perfusion, or any anatomy precluding oncologically safe preservation, prompting conversion to standard RARP. Moreover, two patients meeting the preoperative ≤1.0 cm median lobe criterion were converted intraoperatively to standard RARP because the median lobe (larger than 1cm intraoperatively) distorted the bladder-neck/periurethral plane beyond that compatible with urethra-preserving dissection; this is consistent with the known limited sensitivity of preoperative imaging for the median lobe and with IDEAL stage 2a reporting, under which such intraoperative conversions are prospectively documented and reported without omission. Overall, eight of 47 patients (17%; 95% CI 9–30%) were converted to standard RARP under the predefined intraoperative rule. In robot-assisted urethra-sparing simple prostatectomy, inadvertent urethrotomy requiring intraoperative repair occurred in 26.9% (7/26) and conversion in 3.7% (1/27) of benign cases [44], and a graded series reported full or partial urethral sparing in ~84% with conversion in 16.5% [33]—indicating that complete, uncomplicated urethral preservation is not always achievable even in benign-gland series. Against this background, an 83% completion rate in an early development cohort is within the expected range with completion of the intended procedure in 83% (39/47).

With regard to future IDEAL phase 2b, composite pre-treatment scores such as the Naples Prognostic Score predict biochemical recurrence after RARP [45] and might be adaptable in the stage 2b cohort. Similarly, in future, decision-support systems coupling large language models with surgical world models [46] may help further standardize patient selection, intraoperative decision rules and technique transfer in multicentre evaluation.

Taken together, early outcomes of this IDEAL framework-based cohort support the feasibility and short-term safety of ToP-RAP in a carefully selected patient population, with operative parameters comparable to those of standard RARP and without signals of procedure-specific morbidity. By combining maximal functional-length urethral preservation with intraoperative margin control, ToP-RAP may represent a reproducible strategy to improve early functional recovery while maintaining short-term oncological safety; however, comparative data and longer follow-up are required to define its true functional benefit and oncological non-inferiority.

Several limitations of the present study must be acknowledged. First, the cohort is small, which is consistent with an early IDEAL-stage evaluation and limits generalizability while increasing susceptibility to random error and bias, despite prospective data collection and transparent reporting. Second, ToP-RAP was performed exclusively by high-volume robotic surgeons, which likely reduced technical variability and supported procedural consistency; however, outcomes may not be directly transferable to less experienced operators, and learning-curve effects cannot be reliably inferred from the present dataset. Third, the stringent selection of predominantly low- to intermediate-risk, MRI-visible disease introduces selection bias, and an overestimation of feasibility, safety, and early functional outcomes cannot be excluded. Fourth, the study lacks a comparative control group—such as standard RARP or FT—precluding definitive conclusions regarding incremental functional benefit or oncological non-inferiority. Fifth, in Germany, patients are routinely followed by their referring urologists, with the first standardized postoperative contact (including the first PSA measurement) at approximately 3 months; we therefore synchronized our formal assessments with the 24 h post-catheter-removal, 3-, 6- and 12-month timepoints. We acknowledge as a limitation the absence of a prospectively scheduled, validated instrument (e.g., ICIQ-SF) at exactly 1 week and 1 month. Moreover, early continence was assessed unblinded by pad count and Stamey grade, without pad-weight testing. However, our first assessment was performed 24 h after catheter removal. This is a recognized “immediate continence” endpoint in dedicated early-continence surgical trials and is functionally earlier and more demanding than a 1-week or 1-month assessment, as continence is least stable at this point and improves thereafter [47].

Future prospective evaluation (planned Stage 2b) will incorporate daily pad diaries and ICIQ-SF at these early timepoints to characterize the early-continence advantage more granularly. Sixth, the prostate volume criterion (≤80 mL) served as an upper safety boundary during the learning curve rather than a selection instrument, and the observed median volume of 33 mL (IQR 29–46) reflects the ordinary distribution of the operated population; with only 16 consecutive cases, the absence of glands near the ceiling is a sampling characteristic rather than demonstrable selection bias. While urethral dissection may be technically more demanding in larger glands, the urethra-sparing simple prostatectomy literature indicates that urethral preservation remains feasible in prostates well above 80 mL; the performance of ToP-RAP across a wider volume range should nonetheless be confirmed in the planned stage 2b cohort. Finally, given the short median follow-up, our data document the absence of early biochemical events rather than oncological safety, which cannot be established at this follow-up; longer-term recurrence assessment is essential to evaluate PSA dynamics, to capture late adverse events including urethral healing complications such as ischemic stricture that may arise from periurethral devascularization, and to assess oncological endpoints.

Notwithstanding these limitations, two expert surgeons at two independent centers achieved highly comparable perioperative, functional, and early oncological outcomes, supporting the reproducibility of ToP-RAP within expert practice.

## 5. Conclusions

In this prospective, multicenter IDEAL stage 1–2a/b development study, we introduce ToP-RAP as a urethra-preserving prostatectomy technique in carefully selected men with localized PCa. In keeping with the IDEAL framework, the procedure was conducted with transparent prospective reporting and predefined intraoperative decision rules, including a prespecified conversion strategy to standard RARP whenever urethral preservation or oncological safety could not be ensured. Early perioperative outcomes indicate procedural feasibility and acceptable short-term safety, with no signals of procedure-specific morbidity. The rapid UCR observed supports the underlying premise that preserving the intraprostatic urethra and avoiding vesicourethral anastomosis—when oncologically feasible—may translate into meaningful functional benefit. Stepwise IDEAL-stage evaluation in larger, comparative cohorts with extended follow-up is warranted to define reproducibility, quantify functional advantages, and confirm oncological safety including the assessment of late urethral sequelae.

## Figures and Tables

**Figure 1:**
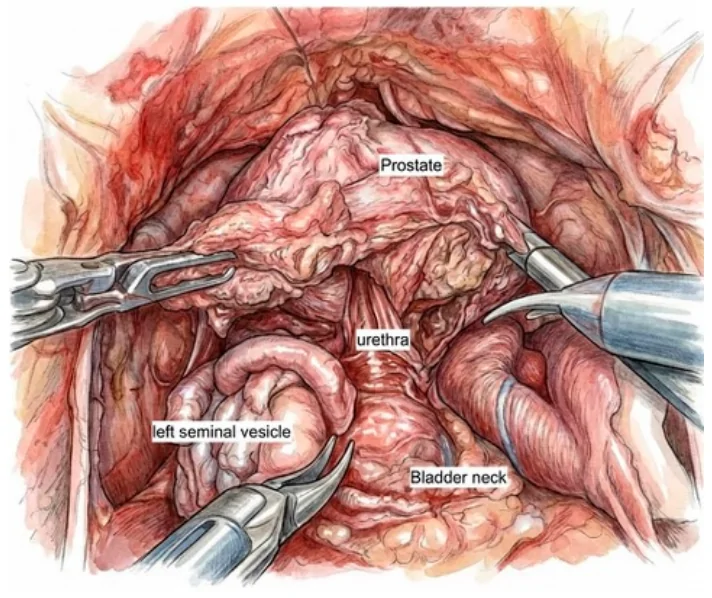
Mobilization of the seminal vesicle with preservation of the surrounding nerves and vessels. After division of the ipsilateral vas deferens, the seminal vesicle and vas deferens were retracted superiorly to expose the ipsilateral prostatic base. The ipsilateral neurovascular bundle was then released in an anterolateral plane from base to apex.

**Figure 2:**
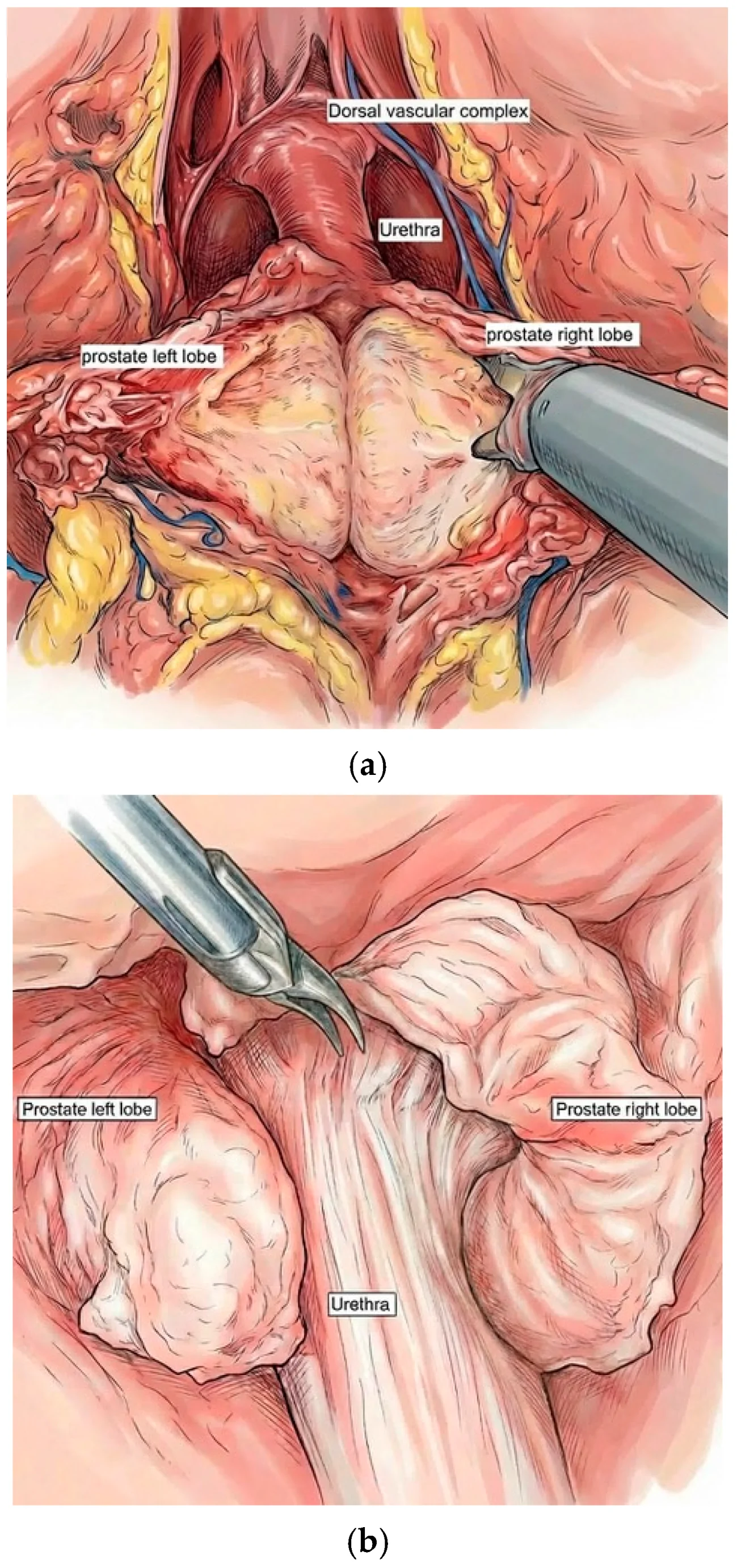
(**a**) Following full exposure of the prostatic apex, an ‘open-book maneuver was performed. The anterior prostate was incised vertically at the 12 o’clock position, allowing the prostatic lobes to be spread laterally. This provided clear visualization’ intraprostatic urethra, ensuring a precise transection at the apical junction. (**b**) The intraprostatic urethra is meticulously dissected from the bilateral prostatic lobes. This plane of dissection reveals the characteristic longitudinal smooth muscle fibers of the urethral wall, ensuring the preservation of maximal urethral thickness.

**Figure 3:**
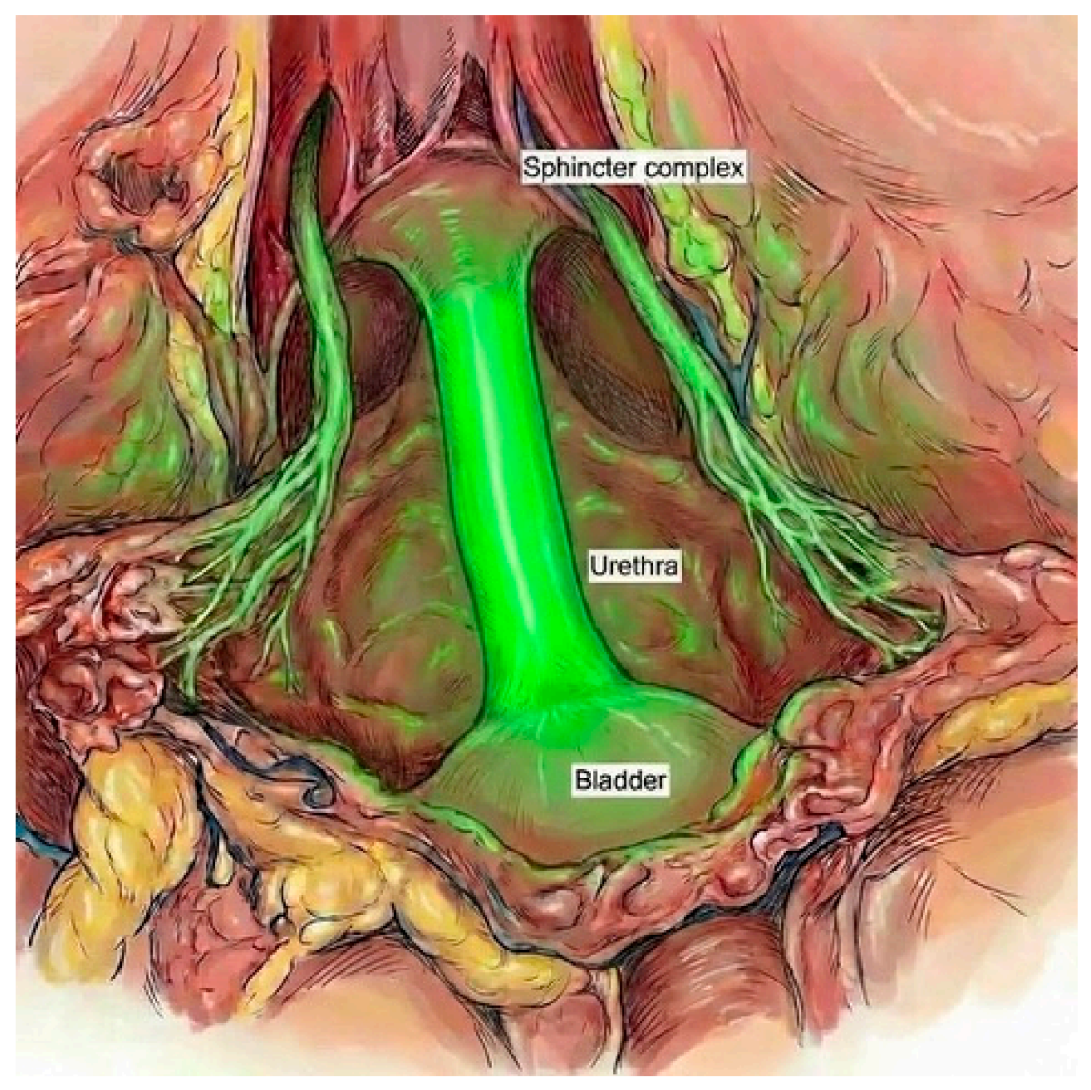
Following a complete removal of the prostate, the vascular integrity of the preserved urethra is assessed using near-infrared fluorescence (NIRF) imaging with Indocyanine Green (ICG). This allowed for real-time visualization of the urethral microcirculation.

**Table 1:** Baseline clinical characteristics of patients undergoing Total en block Urethra Preservation at Robot-assisted Prostatectomy (ToP-RAP).

Variables	Overall (n = 16)
Age, years (median, IQR)	66 (62–71)
Body Mass Index, kg/m^2^ (median, IQR)	27.6 (26–32)
Preoperative PSA, ng/mL (median, IQR)	6.7 (4.7–9.6)
Clinical tumor stage, n (%):	
cT1c	11 (69%)
cT2	4 (25%)
indeterminate	1 (6.3%)
Prostate volume, ml (median, IQR)	33 (29–46)
MRI PIRADS classification, n (%):	
3	2 (13%)
4	9 (56%)
5	3 (19%)
N/A	2 (13%)
Side of MRI lesion, n (%):	
multifocal, bilateral	6 (38%)
unifocal	9 (56%)
indeterminate	1 (6.3%)
Biopsy ISUP grade, n (%)	
1	3 (19%)
2	7 (44%)
3	6 (38%)

**Table 2:** Operative and post-operative characteristics of patients who underwent Total en block Urethra Preservation at Robot-assisted Prostatectomy (ToP-RAP).

	Overall (n = 16)
Console time, minutes (median, IQR)	120 (100–180)
Estimated blood loss, ml (median, IQR)	250 (200–300)
Urethral suturing performed, n (%)	
no	14 (88%)
yes	2 (13%)
Final pathology ISUP grade, n (%)	
1	2 (13%)
2	9 (56%)
3	5 (31%)
Final pathology T stage, n (%):	
pT2	13 (81%)
pT3a	2 (13%)
pT3b	1 (6.3%)
Final pathology N stage, n (%):	
pN0	16 (100%)
Surgical margin status at final pathology, n (%): *	1 (6.3%)
Intra-operative complications according to Clavien–Dindo Classification, n (%)	
CDC3b	1 (6.3%)
Post-operative complications according to Clavien–Dindo Classification (within 90 days), n (%)	
none	16 (100%)

* initially deemed to have negative margin on intraoperative frozen sections, but the margin status was changed on the final conclusive pathology report.

## Data Availability

The data supporting the findings of this study are not publicly available at this time due to privacy and ethical restrictions related to patient-level clinical data. De-identified data may be made available from the corresponding author upon reasonable request and subject to institutional approval.

## Supplementary Materials

The following supporting information can be downloaded at: https://www.mdpi.com/article/10.3390/cancers18193134/s1, Figure S1: CONSORT-style participant flow diagram of the ToP-RAP development study. The flow diagram summarizes enrolment, intraoperative conversion and follow-up for the total cohort of 47 consecutive patients who fulfilled the study criteria, consented to ToP-RAP and underwent surgery with the intention of ToP-RAP at two centers. Eight patients (17%) were converted intraoperatively to standard RARP: urethral preservation was not feasible because of adherent tissue (n = 3), a median lobe that was larger intraoperatively than the ≤1.0 cm preoperative threshold despite meeting that criterion on preoperative imaging (n = 2), or a positive urethral frozen-section margin (n = 3). The remaining 39 patients underwent successful ToP-RAP. Of these, 23 patients with less than 6 months of follow-up at the data cut-off remain in active follow-up and will be reported in subsequent analyses; the 16 patients with at least 6 months of follow-up constitute the analytic cohort and were assessed for perioperative, safety, continence and oncological outcomes. No patient was lost to follow-up. ToP-RAP, Total *en bloc* Urethra Preservation at Robot-assisted Prostatectomy; RARP, robot-assisted radical prostatectomy; Figure S2: Individual PSA trajectories after ToP-RAP (N = 16). Each panel shows one patient’s postoperative PSA values plotted at their time from surgery (x-axis, months, sqrt scale; 6 w, 3 m, 6 m, 12 m, 24 m indicated) on a logarithmic PSA axis (y-axis, ng/mL). Dashed horizontal reference lines mark 0.05 and 0.10 ng/mL. Values ≥ 0.05 ng/mL are shown in red, values < 0.05 ng/mL in blue; circles denote Center A, triangles Center B. Connecting lines are drawn only where ≥ 2 measurements exist; patients with a single measurement are shown as isolated points (no implied trajectory). Six of 16 patients (37.5 %) had at least one PSA ≥ 0.05 ng/mL during follow-up (highest 0.08 ng/mL); no patient reached 0.10 ng/mL at any time point.

## Abbreviations

The following abbreviations are used in this manuscript:

BCR	Biochemical recurrence
BMI	Body mass index
EAU	European Association of Urology
FT	Focal therapy
ICG	Indocyanine green
IDEAL	Idea, Development, Exploration, Assessment, Long-term study
IQR	Interquartile range
ISUP	International Society of Urological Pathology
MRI	Magnetic resonance imaging
mpMRI	Multiparametric magnetic resonance imaging
PCa	Prostate cancer
PET-CT	Positron emission tomography-computed tomography
PI-RADS	Prostate Imaging Reporting and Data System
PSA	Prostate-specific antigen
PSMA	Prostate-specific membrane antigen
RARP	Robot-assisted radical prostatectomy
ToP-RAP	Total *en bloc* Urethra Preservation at Robot-assisted Prostatectomy
UCR	Urinary continence recovery
