# Supplementary material for: Total en Bloc Urethra Preservation at Robot-Assisted Prostatectomy (ToP-RAP): A Prospective IDEAL Stage 1–2a Study
Source: Cancers (Basel). 2026 Sep 28;18(19):3134. doi: 10.3390/cancers18193134 (PMC13664474; doi:10.3390/cancers18193134)
Supplement: Supplementary file 1 [file cancers-18-03134-s001.zip › cancers-4550448-supplementary.pdf]

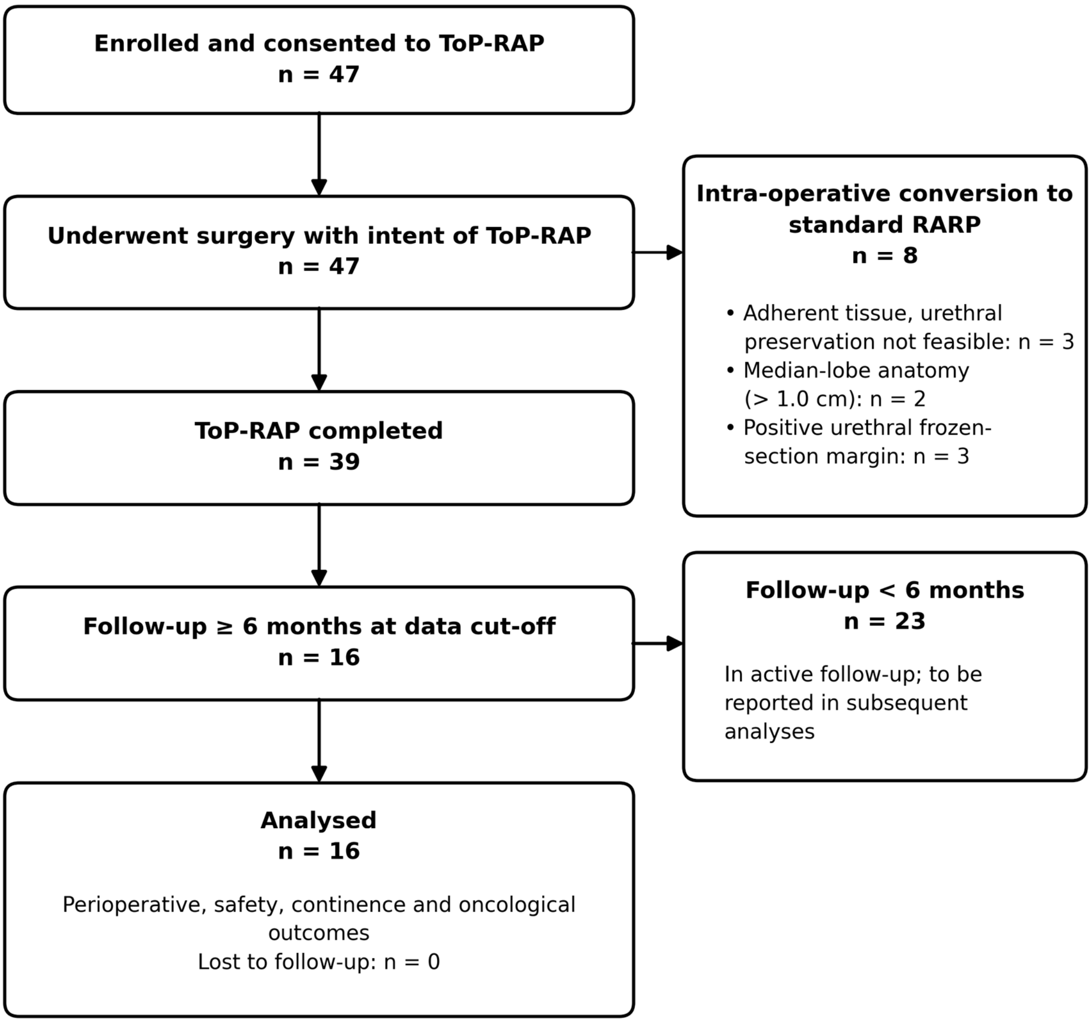

ToP-RAP, Total en bloc Urethra Preservation at Robot-assisted Prostatectomy; RARP, robot-assisted radical prostatectomy.

Supplementary Figure S1. CONSORT-style participant flow diagram of the ToP-RAP development study. The flow diagram summarizes enrolment, intraoperative conversion and follow-up for the total cohort of 47 consecutive patients who fulfilled the study criteria, consented to ToP-RAP and underwent surgery with the intention of ToP-RAP at two centers. Eight patients (17%) were converted intraoperatively to standard RARP: urethral preservation was not feasible because of adherent tissue (n=3), a median lobe that was larger intraoperatively than the ≤1.0 cm preoperative threshold despite meeting that criterion on preoperative imaging (n=2), or a positive urethral frozen-section margin (n=3). The remaining 39 patients underwent successful ToP-RAP. Of these, 23 patients with less than 6 months of follow-up at the data cut-off remain in active follow-up and will be reported in subsequent analyses; the 16 patients with at least 6 months of follow-up constitute the analytic cohort and were assessed for perioperative, safety, continence and oncological outcomes. No patient was lost to follow-up. ToP-RAP, Total en bloc Urethra Preservation at Robot-assisted Prostatectomy; RARP, robot-assisted radical prostatectomy.

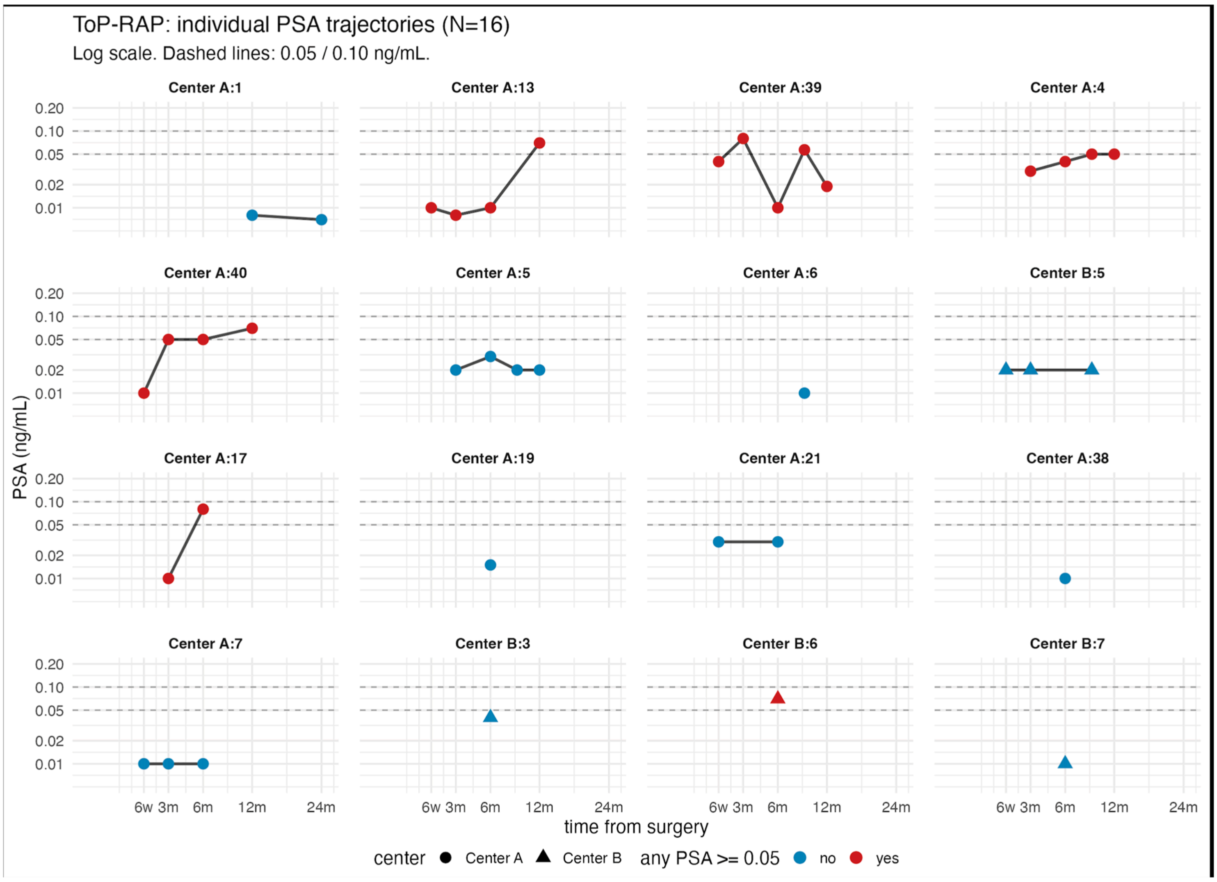

Supplementary Figure S2. Individual PSA trajectories after ToP-RAP (N = 16). Each panel shows one patient's postoperative PSA values plotted at their time from surgery (x-axis, months, sqrt scale; 6 w, 3 m, 6 m, 12 m, 24 m indicated) on a logarithmic PSA axis (y-axis, ng/mL). Dashed horizontal reference lines mark 0.05 and 0.10 ng/mL. Values ≥ 0.05 ng/mL are shown in red, values < 0.05 ng/mL in blue; circles denote Center A, triangles Center B. Connecting lines are drawn only where ≥ 2 measurements exist; patients with a single measurement are shown as isolated points (no implied trajectory). Six of 16 patients (37.5 %) had at least one PSA ≥ 0.05 ng/mL during follow-up (highest 0.08 ng/mL); no patient reached 0.10 ng/mL at any time point.
